# Superspreading Event of SARS-CoV-2 Infection at a Bar, Ho Chi Minh City, Vietnam

**DOI:** 10.3201/eid2701.203480

**Published:** 2021-01

**Authors:** Nguyen Van Vinh Chau, Nguyen Thi Thu Hong, Nghiem My Ngoc, Tran Tan Thanh, Phan Nguyen Quoc Khanh, Lam Anh Nguyet, Le Nguyen Truc Nhu, Nguyen Thi Han Ny, Dinh Nguyen Huy Man, Vu Thi Ty Hang, Nguyen Thanh Phong, Nguyen Thi Hong Que, Pham Thi Tuyen, Tran Nguyen Hoang Tu, Tran Tinh Hien, Ngo Ngoc Quang Minh, Le Manh Hung, Nguyen Thanh Truong, Lam Minh Yen, H. Rogier van Doorn, Nguyen Thanh Dung, Guy Thwaites, Nguyen Tri Dung, Le Van Tan, for the OUCRU COVID-19 research group

**Affiliations:** Hospital for Tropical Diseases, Ho Chi Minh City, Vietnam (N.V.V. Chau, N.M. Ngoc, D.N.H. Man, N.T. Phong, N.T.H. Que, P.T. Tuyen, T.N.H. Tu, L.M. Hung, N.T. Truong, N.T. Dung);; Oxford University Clinical Research Unit, Ho Chi Minh City (N.T.T. Hong, T.T. Thanh, P.N.Q. Khanh, L.A. Nguyet, L.N.T. Nhu, N.T.H. Ny, V.T.T. Hang, T.T. Hien, L.M. Yen, H.R. van Doorn, G. Thwaites, L.V. Tan);; Children’s Hospital 1, Ho Chi Minh City (N.N.Q. Minh);; Centre for Tropical Medicine and Global Health, Oxford, UK (H.R. van Doorn, G. Thwaites);; Ho Chi Minh City Centre for Disease Control and Prevention Ho Chi Minh City (N.T. Dung)

**Keywords:** COVID-19, disease cluster, pandemic, reverse transcription PCR, SARS-CoV-2, superspreading, Vietnam, viruses, whole-genome sequencing, respiratory infections, severe acute respiratory syndrome coronavirus 2, coronavirus disease

## Abstract

We report a superspreading event of severe acute respiratory syndrome coronavirus 2 infection initiated at a bar in Vietnam with evidence of symptomatic and asymptomatic transmission, based on ministry of health reports, patient interviews, and whole-genome sequence analysis. Crowds in enclosed indoor settings with poor ventilation may be considered at high risk for transmission.

Superspreading events occur when a few persons infect a larger number of secondary persons with whom they have contact (*1*,*2*). For severe acute respiratory syndrome coronavirus 2 (SARS-CoV-2), an R_0_ of 2–3 with 6–8 secondary cases has been suggested to constitute a superspreading event (*3*).

Although SARS-CoV-2 is known to be transmitted through droplets and fomites, there has been growing evidence of airborne transmission (*4*,*5*). Better understanding of specific settings in which superspreading events are facilitated remains critical to inform the development and implementation of control measures to avoid future waves of the pandemic (*5*).

On March 18, 2020, a 43-year old man, patient 1, sought treatment at the Hospital for Tropical Diseases in Ho Chi Minh City, Vietnam, for fever, cough, muscle aches, fatigue, and headache. A sample from a nasopharyngeal throat swab specimen taken at admission tested positive for SARS-CoV-2 by reverse transcription PCR.

During the 14 days before the onset of his symptoms on March 17, he had traveled to Thailand and within Vietnam, between Hanoi and Ho Chi Minh City. From 10:00 PM on March 14 until 2:30 AM of the next day, he participated in a St. Patrick’s Day celebration at bar X in Ho Chi Minh City. The bar had 2 indoor areas for clients, an »300-m^2^ area downstairs and an »50-m^2^ area upstairs, with no mechanical ventilation. During open hours, the left and right entrances were typically kept closed to facilitate cooling with air conditioners that recycle indoor air; the middle entrance was kept open. The bar also has naturally ventilated outdoor spaces (Appendix). Patient 1 was inside the bar during the party.

After the confirmed diagnosis of COVID-19 in patient 1, we used contact tracing and testing to detect 18 additional PCR-confirmed cases. Of these, 12 (patients 2–13) were at bar X during the evening of March 14; the other 6 (patients 14–19) were contacts (Table; Appendix [Fig F1]Figure). Of the patients with confirmed cases attending the celebration, 4 were in close contact with patient 1: patients 2–4 went to the celebration with patient 1 and patient 6 worked as a waiter in the bar. Patients 2 and 3, who were roommates, had traveled to Malaysia and returned to Vietnam, patient 2 on March 13 and patient 3 on March 6. The other patients, except for patient 1, had no recent history of travel outside of HCMC (Table).

**Table:** History of travel and patients and contacts of patients positive for severe acute respiratory syndrome coronavirus 2 in cluster associated with bar, Ho Chi Minh City, Vietnam, 2020*

Patient no.	Contact history and epidemiologic factors	Inside bar?	Travel history†	Occupation	Symptom onset	Diagnosed
Patients present at bar X for celebration on March 14–15, 2020						
1	Attended with patients 2, 3, and 4	Y	Y	Pilot	03/17	Mar 18
2	Attended with patients 1, 3, and 4; roommate of patient 3	UNK	Y	Teacher	Unavail.‡	Mar 22
3	Attended with patients 1, 2, and 4; roommate of patient 2	UNK	Y	Teacher	Unavail.	Mar 22
4	Attended with patients 1, 2, and 3	UNK	N	Teacher	Mar 21	Mar 22
5	Attendee; works at shoe company Y	UNK	N	Unavail.	Asympt.	Mar 23
6	Waiter at bar X; in close contact with patient 1	Y	N	Bar X waiter	Mar 16	Mar 23
7	Attendee	UNK	N	Unavail.	Unavail.	Mar 24
8	Attendee; friend of patient 7	UNK	N	Teacher	Unavail.	Mar 24
9	Attendee	UNK	N	Teacher	Mar 25	Mar 26
10	Attendee	UNK	N	Technician	Asympt.	Mar 26
11	Attended with patient 5	UNK	N	Unavail.	Unavail.	Mar 28
12	Attendee	UNK	N	Unavail.	Asympt.	04/02
13	Attended with patient 12	UNK	N	Unavail.	Mar 26	04/03
Contacts of patients present at bar X for celebration on March 14–15, 2020						
14	Contact of patients 5 and 19 as coworkers at shoe company Y	NA	N	Unavail.	Asympt.	Mar 25
15	Household contact of patient 10	NA	N	Unavail.	Asympt.	Apr 1
16	Household contact of patient 6	NA	N	Unavail.	Unavail.	Mar 27
17	Contact (driver) of patients 5 and 14	NA	N	Driver	Mar 27	Mar 30
18	Household contact of patient 14; also contact of patient 5 as a coworker at shoe company Y	NA	N	Unavail.	Asympt.	Mar 30
19	Contact of patients 5 and 14 as coworkers at shoe company Y	NA	N	Unavail.	Unavail.	Apr 6

*Asympt., asymptomatic; NA, Not applicable; unavail., unavailable; UNK, unknown. †Traveled to an area with known local transmission in previous 14 d. ‡Because patient did not enroll in the clinical study, but asymptomatic at time of diagnosis.

**Figure F1:**
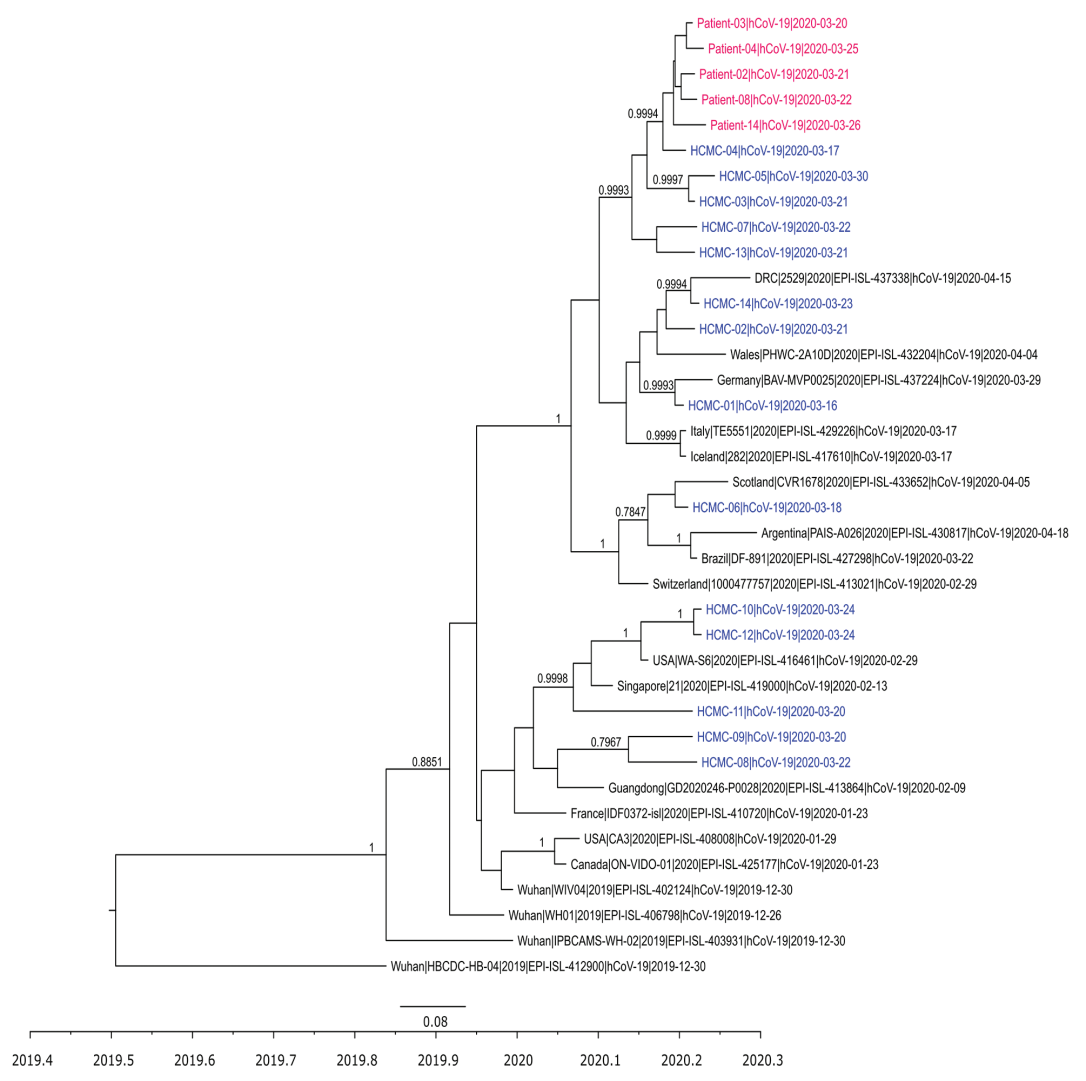
Time-scale phylogenetic tree illustrating the relatedness between whole-genome sequences of severe acute respiratory syndrome coronavirus 2 obtained from patients with confirmed cases of the cluster associated with a bar in Ho Chi Minh City, Vietnam, 2020, and reference sequences. Sequences from the cluster patients are in red; sequences from coronavirus disease patients in Ho Chi Minh City, not related to the cluster, are in blue. For those sequences, we obtained 21 genomes from the remaining 35 patients reported in Ho Chi Minh City as of April 24, 2020, for the purpose of the analysis; subsequently, we used 14 nonidentical sequences for the analysis. Representative sequences from patients not in Vietnam are in black. Posterior probabilities ≥75% are indicated at all nodes. The analysis was carried out using BEAST version 1.8.3 (https://beast.community). For time-scale analysis, only 1 representative of sequences that were 100% identical to each other was included. Whole-genome sequences were generated using ARTIC primers version 3 (ARTIC Network, https://artic.network/ncov-2019).

By exploring the epidemiologic links discovered from in-depth interviews, we identified 3 possible transmission chains involving patients who attended the March 14 celebration (Table; Figure; Appendix Figure). Of these, 2 or 3 patients (patients 5, 10, and possibly 14) were asymptomatic but transmitted SARS-CoV-2 to their contacts (Table; Figure). None of the 19 patients with confirmed cases reported that they had respiratory signs or symptoms on March 14–15. However, in addition to patient 1, a total of 5 others developed mild respiratory symptoms (patient 4 on March 16, patient 6 on March 21, patient 9 on March 25, patient 13 on March 26, and patient 17 on March 27), suggesting an incubation period of 2–12 days. Follow-up data were available for 12 patients who participated in our clinical study (Appendix). Six remained asymptomatic during follow-up (Appendix Table 1).

A total of 11 whole-genome sequences of SARS-CoV-2 were obtained from the patients in the cluster. The obtained sequences were either 100% identical or different from each other by only 1–2 nt (Appendix Table 2). Phylogenetically, they clustered together tightly but were different from sequences obtained from other cases in Ho Chi Minh City during the same period.

As of September 15, 2020, only 30 cases of locally acquired infection had been reported in Ho Chi Minh City (*6*), but this cluster represents the only documented superspreading event (*6*,*7*). Together with data from previous reports (*3*,*8*,*9*), these data suggest that closed settings are facilitators of community transmission of SARS-CoV-2. The mechanism by which infected people without symptoms spread SARS-CoV-2 to others, especially in closed settings, warrants further research, including on transmission through aerosols, which has been suggested (*4*,*10*).

The high level of genome sequence similarity between the SARS-CoV-2 genomes obtained from the patients and the tight clustering on the phylogenetic tree strengthen the epidemiologic link between the PCR-confirmed cases from this cluster. Together with contact history, these data also support transmission chains involving asymptomatic carriers (patients 5 and 14) as the sources of the ongoing infection. However, the identity of the patient in the index case from the bar could not be confirmed, in part because in-depth interview data were available from only 8 of 13 patients with confirmed cases who consented to participate in the study. In conclusion, our results emphasize that persons in crowded indoor settings with poor ventilation may be considered to be at high risk for SARS-CoV-2 transmission.

Appendix. Additional information about the COVID-19 outbreak and response.
