# Supplementary material for: Superspreading Event of SARS-CoV-2 Infection at a Bar, Ho Chi Minh City, Vietnam
Source: Emerg Infect Dis. 2021 Jan;27(1):310–4. doi: 10.3201/eid2701.203480 (PMC7774544; doi:10.3201/eid2701.203480)
Supplement: Supplementary file 1 — Appendix. Additional information about the COVID-19 outbreak and response. [file 20-3480-Techapp-s1.pdf]

Article DOI: https://doi.org/10.3201/eid2701.203480

Superspreading Event of SARS-CoV-2 Infection at a Bar, Ho Chi Minh City, Vietnam

Appendix

The Study

Since the beginning of March 2020, a COVID-19 research program aimed at unraveling the natural history of SARS-CoV-2 infection has been conducted at the Hospital for Tropical Diseases (HTD) and at 1 of its 2 designated COVID-19 centers, Cu Chi Hospital in Ho Chi Minh City, Vietnam (*1*, *2*). These are the largest COVID-19 treatment centers in the southern region, which has a population of >40 million people.

We tested patients admitted to HTD or Cu Chi Hospital using real-time reverse-transcriptase-PCR (rRT-PCR) to confirm SARS-CoV-2 infection. At enrollment, we collected nasopharyngeal throat swabs (NTS) from each participant, and combined them in 1 tube containing 1mL of viral transport medium. We also collected clinical and laboratory data, a travel history, and contact information from each study participant using a case record form developed by the International Severe Acute Respiratory and Emerging Infection Consortium (ISARIC, https://isaric.tghn.org/). The collected NTS sample was placed in storage at 4°C at the study sites within 4 hours and then transferred to the clinical laboratory at HTD for analysis. SARS-CoV-2 detection was carried out using WHO-recommended rRT-PCR assays (*3*).

The clinical studies received approvals from the Institutional Review Board of the HTD and the Oxford Tropical Research Ethics Committee of the University of Oxford. Additionally, we retrieved epidemiologic data from an official COVID-19 website of the Vietnamese Ministry of Health (*4*), and diagnostic results from the HTD database system.

COVID-19 Containment Approach in Vietnam

Since January 2020, various control measures, including isolation of confirmed cases, contact tracing, airport quarantine, and social distancing have been implemented in Vietnam with increasing stringency as the pandemic progressed worldwide (*1*). Accordingly, anyone known to have been in contact with a confirmed COVID-19 case or having traveled to Vietnam from a COVID-19–affected country, were isolated for ≥14 days at a designated isolation center.

From the second week of March 2020, all isolated persons were subject to serial SARS-CoV-2 NTS screening by rRT-PCR at least 2X (day 1 and day 14 of the quarantine) (*1*). A confirmed case was established if 2 independent RT-PCR assays (E gene and RdRP RT-PCR assays) were positive.

In response to escalation of COVID-19 cases in Vietnam, on April 1, 2020 the government issued Directive #16, requiring mass masking, was applied throughout Ho Chi Minh City. This directive was lifted on April 23, 2020.

Description of bar X

The bar has both indoor and outdoor spaces. The majority of the bar space, ≈350 m^2, is indoors. The ground floor has an indoor space ≈300 m^2 and outdoor spaces of ≈100 m^2 at the front with limited seating. At the back of the bar is a small outdoor smoking section of ≈20 m^2. The upstairs area is divided into an indoor area and a balcony. The former is ≈50 m^2, with a pool table, a small bar, and some seating; the latter is ≈20 m^2.

When the bar is open, the front is partly open, allowing some natural ventilation, but has air conditioners that recycle indoor air throughout. The other two entrances to the bar are kept closed to retain cool air. Except for some of the bar staff, none of the >200 participants was wearing masks on the night of the celebration.

OUCRU COVID-19 Research Group

Hospital for Tropical Diseases, Ho Chi Minh City, Vietnam: Nguyen Van Vinh Chau, Nguyen Thanh Dung, Le Manh Hung, Huynh Thi Loan, Nguyen Thanh Truong, Nguyen Thanh Phong, Dinh Nguyen Huy Man, Nguyen Van Hao, Duong Bich Thuy, Nghiem My Ngoc, Nguyen Phu Huong Lan, Pham Thi Ngoc Thoa, Tran Nguyen Phuong Thao, Tran Thi Lan Phuong, Le Thi Tam Uyen, Tran Thi Thanh Tam, Bui Thi Ton That, Huynh

Kim Nhung, Ngo Tan Tai, Tran Nguyen Hoang Tu, Vo Trong Vuong, Dinh Thi Bich Ty, Le Thi Dung, Thai Lam Uyen, Nguyen Thi My Tien, Ho Thi Thu Thao, Nguyen Ngoc Thao, Huynh Ngoc Thien Vuong, Pham Ngoc Phuong Thao, Phan Minh Phuong

Oxford University Clinical Research Unit, Ho Chi Minh City, Vietnam: Dong Thi Hoai Tam, Evelyne Kestelyn, Donovan Joseph, Ronald Geskus, Guy Thwaites, H. Rogier van Doorn, Ho Van Hien, Huynh Le Anh Huy, Huynh Ngan Ha, Huynh Xuan Yen, Jennifer Van Nuil, Jeremy Day, Joseph Donovan, Katrina Lawson, Lam Anh Nguyet, Lam Minh Yen, Le Nguyen Truc Nhu, Le Thanh Hoang Nhat, Le Van Tan, Sonia Lewycka Odette, Louise Thwaites, Maia Rabaa, Marc Choisy, Mary Chambers, Motiur Rahman, Ngo Thi Hoa, Nguyen Thanh Thuy Nhien, Nguyen Thi Han Ny, Nguyen Thi Kim Tuyen, Nguyen Thi Phuong Dung, Nguyen Thi Thu Hong, Nguyen Xuan Truong, Phan Nguyen Quoc Khanh, Phung Le Kim Yen, Sophie Yacoub, Thomas Kesteman, Nguyen Thuy Thuong Thuong, Tran Tan Thanh, Tran Tinh Hien, Vu Thi Ty Hang

References

1. Chau NVV, Thanh Lam V, Thanh Dung N, Yen LM, Minh NNQ, Hung LM, et al.; OUCRU COVID-19 research group. The natural history and transmission potential of asymptomatic SARS-CoV-2 infection. Clin Infect Dis. 2020;ciaa711. PubMed https://doi.org/10.1093/cid/ciaa711

2. Van Tan L, Thi Thu Hong N, My Ngoc N, Tan Thanh T, Thanh Lam V, Anh Nguyet L, et al.; for OUCRU COVID-19 research group. SARS-CoV-2 and co-infections detection in nasopharyngeal throat swabs of COVID-19 patients by metagenomics. J Infect. 2020;81:e175–7. PubMed https://doi.org/10.1016/j.jinf.2020.06.033

3. Corman VM, Landt O, Kaiser M, Molenkamp R, Meijer A, Chu DK, et al. Detection of 2019 novel coronavirus (2019-nCoV) by real-time RT-PCR. Euro Surveill. 2020;25. PubMed https://doi.org/10.2807/1560-7917.ES.2020.25.3.2000045

4. Ministry of Health of Vietnam. Updated information about COVID-19 pandemic. Official page on acute respiratory infections COVID-19 [cited 2020 Sep 5]. http://ncov.moh.gov.vn

Appendix Table 1. Demographics and admission clinical and laboratory data of the study participants

Demographics	(N = 19)
Age in years, median (range)	33 (22–51)
Gender (female/male), n/n	9/10
Nationality	n (%)
Vietnamese	5 (26)
Others	14 (74)
Admission clinical and laboratory data	N = 12*
Days from confirmed diagnosis to enrolment, median (range)	2 (1 – 4)
Days from admission to enrolment, median (range)	1 (0 – 2)
Laboratory results	Median (range)/normal range
White-cell count (×10^3/µL)	5.5 (3.2–6.9)/(4–11)
Lymphocyte counts (×10^3/µL)	1.8 (0.5–2.6)/(1.5–4)
Hemoglobin (g/dl)	14.8 (10–17.3)/13–18)
Hematocrit (%)	39.5 (29–43.4)/(37–52)
Platelet count (per µL)	243 (166–327)/(150–450)
Glucose (mg/liter)	89.7 (64 –340)/(70–130)
Creatinine (mg/dl)	1.0 (0.9–1.5)/(0.5–1.2)
Aspartate aminotransferase (U/L)	24.2 (17–56.8)/(<40)
Alanine aminotransferase (U/L)	25.4 (11.5–44.9)/(<37)
Clinical signs/symptoms	n (%)
Fever,	3 (25)
Cough, n (%)	10 (33)
Rhinorrhea, n (%)	4 (33)
Fatigue, n (%)	2 (17)
Diarrhea, n (%)	0
Sore throat, n (%)	1 (8)
Muscle pain, n (%)	2 (17)
Headache, n (%)	2 (17)
Abdominal pain, n (%)	0
Lost sense of smell, n (%)	0
Baseline comorbidity, n (%)	4 (33)†

*Only available from patients participating in the aforementioned study

†Obesity (n = 2), diabetes without complication (n = 1), and kidney associated problem with 85% reduction of kidney function (n = 1)

Appendix Table 2: The number of nucleotides different among the obtained genome sequences of the cluster

Patient*	17	18	5†	1†	16	3†	6†	14	4†	8†	2†
17	NA	0	0	0	0	0	0	1	1	1	1
18	0	NA	0	0	0	0	0	1	1	1	1
5†	0	0	NA	0	0	0	0	1	1	1	1
1†	0	0	0	NA	0	0	0	1	1	1	1
16	0	0	0	0	NA	0	0	1	1	1	1
3†	0	0	0	0	0	NA	0	1	1	1	1
6†	0	0	0	0	0	0	NA	1	1	1	1
14	1	1	1	1	1	1	1	NA	2	2	2
4†	1	1	1	1	1	1	1	2	NA	2	2
8†	1	1	1	1	1	1	1	2	2	NA	2
2†	1	1	1	1	1	1	1	2	2	2	NA

NA, not applicable

*Patient numbers correspond to those in Figure 1 and in the text

†Attended celebration on March 14, 2020

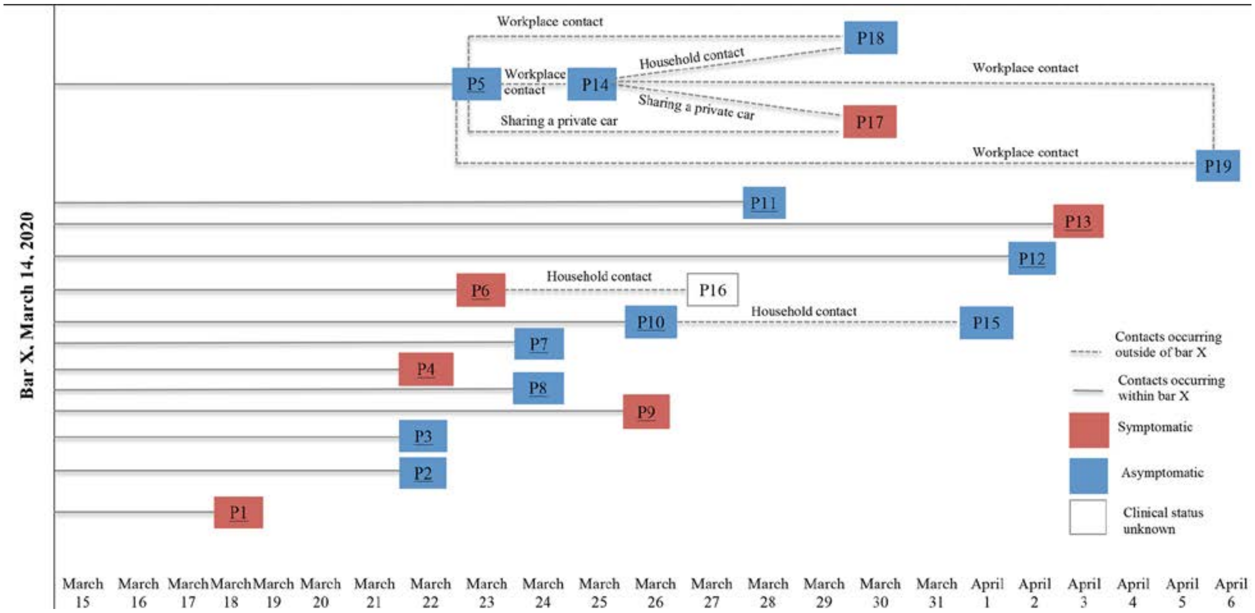

Appendix Figure. Illustration of timeline of the investigation and potential contacts occurring between SARS-CoV-2 positive cases. Details about travel history and contacts between cases, and types of workplace are shown in the Table in the main text. Dates shown are when a confirmed diagnosis was established. Patients 2, 3, 7, 8, 11, 16, and 19 did not consent to participate in the clinical study. Patients 2, 3, 7, 8, 11, 11, and 19 were asymptomatic at diagnosis. Patients in blue boxes were all asymptomatic during the course of quarantine. Clinical status of patient 16 was unknown. Patients from the initial bar cluster (1–13) are underlined.
